# Vivax malaria and bacteraemia: a prospective study in Kolkata, India

**DOI:** 10.1186/1475-2875-12-176

**Published:** 2013-05-31

**Authors:** Sujit Kumar Bhattacharya, Dipika Sur, Shanta Dutta, Suman Kanungo, R Leon Ochiai, Deok Ryun Kim, Nicholas M Anstey, Lorenz von Seidlein, Jacqueline Deen

**Affiliations:** 1 Society for Applied Studies, Kolkata, India; 2 National Institute of Cholera and Enteric Diseases, Kolkata, India; 3 International Vaccine Institute, Seoul, Korea; 4 Menzies School of Health Research and Charles Darwin University, Rocklands Drive, Casuarina, NT 0810, Australia

**Keywords:** Bacteraemia, Falciparum malaria, Vivax malaria

## Abstract

**Background:**

Falciparum malaria increases the risk for bacteraemia, whereas the relationship between vivax malaria and bacteraemia is not clear. Data from a prospective fever surveillance study in Kolkata, India were reanalysed for the potential association between *Plasmodium vivax* malaria and bacteraemia.

**Methods:**

Patients of all ages presenting with fever of three days or more to a project health outpost were invited to participate. A blood film and blood culture was performed on presentation. Treatment and referral were provided according to national guidelines. The case fraction and incidence of malaria, bacteraemia, and co-infection were calculated.

**Results:**

3,371 participants were enrolled during a one-year study period, of whom 93/3,371 (2.8%) had malaria (89/93 [95.7%] *Plasmodium vivax*) and 256 (7.6%) bacteraemia. There were 154 malaria, 423 bacteraemia and 10 *P. vivax*-bacteremia coinfection episodes per 100,000/year. Among the malaria-bacteraemia co-infections, all were vivax malaria and 5/6 (83%) bacteria isolated were Gram-negative (one *S.* Typhi, one *S.* Paratyphi A, three other Gram-negative). Bacteraemia occurred in 6/89 (6.7% [95%CI: 3.1-13.9%]) of *P. vivax* cases versus 250/3,278 (7.6% [95% CI: 6.7-8.6%]) without *Plasmodium* infection (p=0.76).

**Conclusions:**

While an increased risk was not demonstrated, concomitant bacteraemia occurs frequently in vivax malaria in an area with a high background incidence of bacteraemia, and should be considered in cases of vivax malaria with severe manifestations.

## Background

The association between *Plasmodium falciparum* infection and bacteraemia is well documented [1]. A report from Nigeria suggested the association between invasive salmonellosis and falciparum malaria in children [2]. It was then shown that young Gambian children with invasive non-typhoidal *Salmonella* infection were more anaemic and more likely to have evidence of recent malaria than were children of the same age with other forms of septicaemia [3]. Studies in Kenya showed that an acute episode of falciparum malaria predisposes to bacteraemia and that the latter contributes to the clinical picture of severe malaria [4]. The association between non-typhoidal *Salmonella* bacteraemia and severe anaemia has also been found in Malawi [5,6]. A recent study in Kenya has provided the strongest evidence to date of *P. falciparum* infection increasing the risk for bacteraemia, particularly for Gram-negative invasive infections [7]. Strategies that control falciparum malaria reduce child mortality to a greater extent than can be attributed to malaria alone [8,9], likely through its mitigation of bacterial disease.

In contrast to falciparum malaria, the relationship between vivax malaria and bacteraemia has been less explored. The rate of concomitant bacteraemia in severe *Plasmodium vivax* malaria is not known, nor the extent to which concomitant bacterial infections contributes to the manifestations of severe vivax disease. Although there are reports of uncomplicated and severe vivax malaria with concurrent bacteraemia [10-12], recent series of severe vivax malaria cases with sepsis-like syndromes, including shock and multi-organ dysfunction, have not included systematic collection of pre-antibiotic blood cultures [13-15]. Importantly, there are increasing reports of *P. vivax* with severe clinical syndromes [16]. Data from a prospective fever surveillance study was reanalysed to assess the frequency of concomitant bacteraemia in *P. vivax*-infected patients presenting for primary care treatment of fever and the possible association between these two conditions.

## Methods

### Study population and procedures

The study population and procedures have been previously described in detail [17]. Briefly, the site was in Kolkata, India and consisted of wards 29 and 30, which are legally registered urban slum areas (*bustees)*. The area is densely populated with narrow streets, little space between houses, intermittent piped water supply and inadequate sanitation facilities. A 2004 study census recorded a population of 60,452, of whom 15,980 were two months to 14 years of age [17]. Prospective fever surveillance was conducted from January-December 2004 in preparation for a large typhoid fever vaccine effectiveness trial [18]. Five project health outposts set-up in the study area were open daily from 08:00–20:00 hours. Two project health outposts in the city’s infectious diseases hospital and children’s referral hospital were open 24 hours a day. Each outpost, operated by a physician and five assistants, offered free treatment according to national guidelines or referral with free transport to hospital when required. From the study population, all patients with fever for three days or more, irrespective of age, were invited to participate.

From each participant, a drop of blood was used for a thin blood film and stained with Leishman’s stain. At least 100 high-power thin film microscopic fields were examined to exclude the diagnosis of malaria. *Plasmodium* species were identified on the thin film and confirmed by a senior malariologist. Approximately 5–8 ml of blood from adults and 3–5 ml from children were collected and used to inoculate BACTEC-Plus Aerobic® and BACTEC-Peds Plus® culture vials (Becton Dickinson), respectively. Inoculated Bactec bottles were incubated at 37°C for seven days with subcultures made on MacConkey agar (Difco) during the 1st, 2nd, 4th, and 7th days of incubation. Colonies detected on the plates were checked by Gram staining and other biochemical tests following standard procedures. Identification of *Salmonella enterica* serotype Typhi and Paratyphi strains was confirmed by slide and tube agglutination using polyvalent and monovalent factor antisera (Becton Dickinson) for O and H antigens. *Salmonella* isolates were preserved in glycerol stock at −70°C, and their identities verified at a reference laboratory (University of Oxford-Wellcome Trust Clinical Research Unit, Ho Chi Minh City, Vietnam). Other Gram-negative and Gram-positive bacteria were not identified further.

### Data management, definitions and analysis

Standard case report forms were used to record medical history, physical examination findings, laboratory results and clinical management. Data were double-entered into custom-made data entry programs (FoxPro, Microsoft Corp) including error, range and consistency check programs.

Fever was defined as history of a rise in body temperature as recalled by the patient/caregiver or presence of axillary temperature ≥37.5°C on presentation. Only one blood culture was performed (on enrolment). Bacterial isolates were classified as Gram-negative (including *Salmonellae*) or Gram-positive. Malaria was defined as fever with the presence of asexual malaria parasites on the blood film, classified as *P. falciparum, P. vivax, Plasmodium malariae* or *Plasmodium ovale*. Co-infection was defined as the presence of bacteraemia and malaria.

The one-year incidence (per 100,000 population) of fever episodes, malaria, bacteraemia and co-infections was estimated using the number of such cases residing in the referral population as the numerator and the age-specific population from a study census as the denominator. The incidence of *P. vivax* malaria and typhoid fever have been previously reported [17], but not of other bacteraemia and co-infections. The potential association between the two binary variables, *P. vivax* parasitaemia and bacteraemia on presentation for treatment, was assessed using the Chi square test. Data analysis was performed using Stata software, version 11.0. Statistical significance was designated at p value <0.05.

### Ethics

Written informed consent was obtained from each patient or his/her parent or guardian prior to participation. The Health Ministry Screening Committee of the Government of India, the Institutional Review Board of the International Vaccine Institute (Seoul, Korea) and the Secretariat Committee for Research Involving Human Subjects, World Health Organization (Geneva, Switzerland) approved the study. The confidentiality of information collected from study participants, including those derived from clinical specimens was ensured during and after the conduct of the study. All participant data were computerized using password protection and all hard copy records were kept at a secure place and available only to authorized study personnel for research purposes.

## Results

During the one-year study period, 3,371 (1,234 who were 2 months to 14 years of age) were enrolled and included in the analysis (Table 1). Malaria parasites were detected in the blood of 93/3,371 (2.8%) participants. Of the malaria cases, 89/93 (95.7%) were *P. vivax*, 2 (2.2%) *P. falciparum*, one (1.1%) *P. malariae* and one (1.1%) unspecified. Bacteraemia was detected in 256/3,371 (7.6%) participants. Of the 256 bacterial isolates, 223 (87.1%) were Gram-negative, including *S.* Typhi (95 or 42.6%), *S.* Paratyphi A (63 or 28.3%) and other *Salmonellae* (1 or 0.4%). Of the 89 vivax malaria cases 6 (6.7% [95%CI: 3.1-13.9%]) had concomitant bacteraemia but similarly, 250/3278 (7.6% [95% CI: 6.7-8.6%]) of the non-malarious cases were also bacteraemia (p=0.76).

**Table 1:** Number (incidence per 100,000 population) of malaria, bacteraemia and co-infections

	**All ages**	**Children 2 months to 14 years**
Population denominator	60,452	15,980
No (%) participants enrolled	3,371 (5.6)	1,234 (7.7)
Mean age of participants	24.7	7.7
No (%) female participants	1,641 (48.7)	591 (47.9)
**Number (incidence per 100,000 population) of:**		
Malaria	93 (154)	21 (131)
Bacteraemia	256 (423)	114 (713)
Gram-positive bacteraemia	32 (53)	4 (25)
Gram-negative bacteraemia	223 (369)	109 (682)
Typhoid fever	95 (157)	57 (357)
Other Salmonellosis (63 *S.* Paratyphi A and 1 other)	64 (106)	31 (194)
**Number (incidence per 100,000 population) of co-infections:**		
Malaria & bacteraemia	6 (10)	2 (13)
Falciparum malaria & bacteraemia	0 (0)	0 (0)
Vivax malaria & bacteraemia	6 (10)	2 (13)
Malaria & Gram-positive bacteraemia	1 (2)	0 (0)
Malaria & Gram-negative bacteraemia	5 (8)	2 (13)
Malaria & typhoid fever	1 (2)	1 (6)
Malaria & paratyphoid fever	1 (2)	0 (0)

There were 154 malaria and 423 bacteraemia episodes per 100,000 population (131 and 713 in children) per year (Table 1). Malaria and bacteraemia co-infections occurred at 10 per 100,000 population (13 in children) per year. Among the malaria-bacteraemia co-infections, all were vivax malaria and 5/6 (83%) bacteria isolated were Gram-negative (one *S.* Typhi, one *S.* Paratyphi A, three other Gram-negative). There was one vivax malaria and two typhoid fever cases who were hospitalized. None of the patients with concomitant malaria and bacteraemia were hospitalized. There were no deaths among the enrolled participants.

## Discussion

In this Indian study site, a high proportion (6.7%) of vivax malaria cases presenting for treatment had concomitant bacteraemia. With 7.6% of non-malarious cases also bacteraemic, it could not be demonstrated that *P. vivax* infection increases the risk of bacteraemia. Nevertheless, in areas like Kolkata, with a high incidence of both vivax malaria and bacteraemia, co-infections are common.

The high frequency of *P. vivax*-bacteraemia co-infection in this Indian site is notable because a number of series from across this region have reported *P. vivax* infection with severe sepsis-like syndromes [14,16]. Bacterial co-infections could contribute to severe sepsis-like presentations reported with *P. vivax* parasitaemia [10-16]. In falciparum malaria, bacteraemia, malnutrition and HIV infection are biologically associated with severe disease and an increased risk of death, rather than just being alternative diagnoses in coincidentally parasitized patients [19]. Similar relationships are plausible in vivax malaria where severe disease may arise from *P. vivax* infection, bacterial infection, or an additive or synergistic combination of both, often with other comorbidities [16]. Hypnozoite activation by significant systemic illness leading to vivax relapse has also been proposed [20].

The study has several limitations. The confidence interval for the 6.7% frequency of *P. vivax*-bacteraemia co-infection is wide (3.1-13.9%); an increased risk of bacteraemia from *P. vivax* cannot be excluded. Considering the insensitivity of blood cultures and widespread community antibiotic usage, the frequency of bacteraemia and bacteremia-*P. vivax* coinfection may be higher. Second, since both malaria and bacteraemia were diagnosed and treated at the same time (on presentation), only the rates of concurrent vivax malaria and bacteraemia and a potential association between both could be assessed and not whether the former predisposes to the latter or vice versa. And third, since majority of cases were captured in the community and treated early, cases with severe manifestations were uncommon with only three patients hospitalized. Despite these limitations, this study provides the largest and best available population-based dataset on the relationship between *P. vivax* infection and bacteraemia.

The relationship between *P. vivax* malaria and bacteraemia is important to understand. *P. vivax* is a major cause of morbidity and mortality and is estimated to comprise around 40% of the world’s malaria burden [21]. As current malaria control and elimination programmes disproportionately reduce the incidence of *P. falciparum*, the global fraction of malaria due to *P. vivax* is expected to rise. Whether *P. vivax* increases the risk for bacteraemia, the extent to which concurrent bacterial infection contributes to manifestations and outcomes of severe disease in *P. vivax* infection and whether systemic bacterial infections increase vivax relapses all require further elucidation. Multi-country, hospital-based studies with systematic, quality-assured pre-antibiotic blood and other sterile-site cultures in vivax malaria patients, especially those with severe manifestations, are needed, as well as follow-up studies of bacteraemic patients to evaluate the risk of vivax relapse.

## Conclusions

In a region with a high risk of bacterial infection, concomitant bacteraemia in *P. vivax* infection is common. Severe sepsis-like syndromes occurring in association with *P. vivax* infection require blood cultures and consideration of empiric broad-spectrum antimicrobial treatment. Further studies are needed to understand the interaction between vivax malaria and bacteraemia.

## Competing interests

The authors declare that they have no competing interests.

## Authors’ contributions

Design and implementation of the field studies: SKB, DS, SK, RLO, JD and LvS. Laboratory supervision: SD. Conception of the current analysis: NA. Design of current analysis: LvS and JD. Statistical analysis: DRK. Wrote the first draft: NA, LvS and JD. Participated in the revision of the manuscript: SKB, DS, SD, RLO, DRK, NA, LvS, and JD. All authors read and approved the final manuscript.
